# Testing Lead’s Limits: Time for Another Reassessment of Guidelines?

**Published:** 2008-02

**Authors:** M. Nathaniel Mead

Cohort data during the 1980s linked blood lead levels of at least 10 μg/dL with low cognitive test scores in children, prompting the decision by the Centers for Disease Control and Prevention to redefine the action level for elevated blood lead from 25 to 10 μg/dL. Now, new data add to the growing evidence that the 10-μg/dL level may not be protective **[*EHP* 116:243–248; Jusko et al.]**.

The investigators recruited children aged 24–30 months who had been previously enrolled in a dust control study. All the children were born between July 1994 and January 1995 and lived in Rochester, New York, with parents expressing no plans to relocate. To reduce the possibility of misclassification of exposure, blood samples were collected for measuring blood lead on up to 8 occasions (at ages 6, 12, and 18 months, and annually from age 2 through 6 years).

The children were given the Wechsler Preschool and Primary Scale of Intelligence during their 6-year visit by an examiner trained in neurobehavioral testing and blinded to each child’s blood lead level. These assessments were made at an age when IQ is measured reliably and is a significant predictor of IQ scores and educational and occupational success during adolescence and adulthood. The data analysis employed a regression model that controlled for family income; maternal education, race, prenatal smoking, and Stanford-Binet IQ score; child’s birth weight; breastfeeding; crowding in the home; and quality of childrearing (using the Home Observation for Measurement of the Environment Inventory).

The average blood lead level was 7.2 μg/dL, and lead concentrations for more than half the children never exceeded the 10 μg/dL mark. Even at these concentrations, blood lead levels were inversely related to IQ scores. The association was most pronounced for the Full-Scale and Performance IQ scores. Children whose blood lead levels measured in the 5- to 9.9-μg/dL range had significantly lower IQ scores than children with levels below 5 μg/dL. A descriptive analysis of peak exposure throughout early childhood suggested an inverse association between maximal blood lead level and IQ at blood lead levels less than 3 μg/dL; levels as low as about 2 μg/dL were associated with significant IQ declines. These findings, reinforced by previous data gathered by the same research team, support the need for a further reassessment of standard guidelines for responding to blood lead in infants and children.

